# Short-term fasting accompanying chemotherapy as a supportive therapy in gynecological cancer: protocol for a multicenter randomized controlled clinical trial

**DOI:** 10.1186/s13063-020-04700-9

**Published:** 2020-10-15

**Authors:** Daniela Koppold-Liebscher, Christian S. Kessler, Nico Steckhan, Vanessa Bähr, Cornelia Kempter, Manfred Wischnewsky, Marisa Hübner, Barbara Kunz, Marion Paul, Stefanie Zorn, Sophia Sari, Michael Jeitler, Rainer Stange, Andreas Michalsen

**Affiliations:** 1 Institute of Social Medicine, Epidemiology and Health Economics, Charité – Universitätsmedizin Berlin, Corporate Member of Freie Universität Berlin, Humboldt-Universität zu Berlin, and Berlin Institute of Health, Berlin, Germany; 2 Department of Internal and Integrative Medicine, Immanuel Hospital Berlin, Berlin, Germany; 3 Department of Gynecology, Waldfriede Hospital, Berlin, Germany; 4 Department of Mathematics and Computer Science, University of Bremen, Bremen, Germany; 5 Vivantes Hospital Breast Centre, Berlin, Germany; 6 Department of Medicine I, Section of Clinical Nutrition and Dietetics, Medical Center – University of Freiburg, Freiburg im Breisgau, Germany; 7 Department of Nutrition, Technische Universität München, Munich, Germany

**Keywords:** Breast cancer, Ovarian cancer chemotherapy, Short-term fasting, Intermittent fasting, Plant-based nutrition, Caloric restriction, Randomized controlled trial

## Abstract

**Background/objectives:**

A few preliminary studies have documented the safety and feasibility of repeated short-term fasting in patients undergoing chemotherapy. However, there is a lack of data from larger randomized trials on the effects of short-term fasting on quality of life, reduction of side effects during chemotherapy, and a possible reduction of tumor progression. Moreover, no data is available on the effectiveness of fasting approaches compared to so-called healthy diets. We aim to investigate whether the potentially beneficial effects of short-term fasting can be confirmed in a larger randomized trial and can compare favorably to a plant-based wholefood diet.

**Methods:**

This is a multicenter, randomized, controlled, two-armed interventional study with a parallel group assignment. One hundred fifty patients, including 120 breast cancer patients and 30 patients with ovarian cancer, are to be randomized to one of two nutritional interventions accompanying chemotherapy: (1) repeated short-term fasting with a maximum energy supply of 350–400 kcal on fasting days or (2) repeated short-term normocaloric plant-based diet with restriction of refined carbohydrates. The primary outcome is disease-related quality of life, as assessed by the functional assessment of the chronic illness therapy measurement system. Secondary outcomes include changes in the Hospital Anxiety and Depression Score and as well as frequency and severity of chemotherapy-induced side effects based on the Common Terminology Criteria of Adverse Events. Explorative analysis in a subpopulation will compare histological complete remissions in patients with neoadjuvant treatments.

**Discussion/planned outcomes:**

Preclinical data and a small number of clinical studies suggest that repeated short-term fasting may reduce the side effects of chemotherapy, enhance quality of life, and eventually slow down tumor progression. Experimental research suggests that the effects of fasting may partly be caused by the restriction of animal protein and refined carbohydrates. This study is the first confirmatory, randomized controlled, clinical study, comparing the effects of short-term fasting to a short-term, plant-based, low-sugar diet during chemotherapy on quality of life and histological tumor remission.

**Trial registration:**

ClinicalTrials.gov NCT03162289. Registered on 22 May 2017

## Background

In recent years, short-term fasting (STF) as a distinct form of caloric restriction has been extensively investigated, particularly in animal experiments, but also in some clinical studies. So far, results point to favorable effects of STF in preventive and therapeutic terms on degenerative, immunological, inflammatory, and metabolic diseases: findings are consistent across all species [1–7]. Primates and rodents show a reduced risk of developing cancer if they are exposed to STF [1, 8]. Clinical studies on STF in humans are rare, and different definitions of short-term or intermittent fasting are being used [9–11]. Until now, there has been no unanimously agreed standard definition of either short-term or intermittent fasting. As one of the worldwide leading research centers for clinical fasting, we use the term “short-term fasting” to refer to fasting periods exceeding typical intermittent fasting or time-restricted eating ranging from 12 to 48 h. More specifically, we use the term STF in our study to define a subtotal caloric restriction of 60–72 h, as we did in a previous publication [12]. Thus, in our definition, intermittent fasting differs from STF mainly in its duration.

For cancer patients, chemotherapy is usually perceived as a strong physical and psychological burden and can cause acute and long-term side effects. Chemotherapy can even be discontinued due to the severity of its side effects, thereby not attaining the goals of reducing tumor size or eliminating the tumor. With the exception of the administration of certain drugs (e.g., antiemetics [13, 14]) and therapeutic exercise [15, 16], only a few interventions are known to help reduce chemotherapy-induced side effects [17]. Hence, there is an evident need for additional interventions that may improve the tolerance to chemotherapy.

Experimentally, it has been shown that short-term fasting induces a “protective mode” in healthy cells against chemotherapeutic toxicity while it enhances the susceptibility of tumor cells to chemotherapy [18]. This differential effect of fasting on cell susceptibility in the situation of chemotherapy has been described as “differential stress resistance” (DSR) [19, 20]. In healthy cells, fasting triggers distinct protective metabolic and gene expression changes, such as upregulation of DNA repair mechanisms, autophagy, and downshift of metabolic pathways including IGF-1-dependent signaling [21]. This not only limits cell growth and proliferation, but also activates the signaling pathways to protect healthy cells against reactive oxygen species (ROS) produced by chemotherapeutics. Cancer cells, on the other hand, are largely independent of proliferative signals and insensitive to anti-proliferative signals [18]. As a consequence, cancer cells are insufficiently able to adapt to reduced concentrations of glucose, growth factors, and other signals caused by fasting and caloric restriction and are therefore more susceptible to chemotherapy [22]. Since STF is not associated with long-term weight loss [23], which can significantly worsen the prognosis of cancer patients [24, 25], it has been evaluated in early pilot studies as a potential supportive treatment in chemotherapy [23]. Experimentally, the combination of fasting and chemotherapy appears to be more effective than chemotherapy alone regarding efficacy and tolerability, particularly in breast cancer [4, 18, 26].

In a case series on patients with a variety of malignancies, beneficial effects of STF were documented; the feasibility of STF and a reduction of the severity of chemotherapy-induced side effects were shown [27]. The effects of STF were investigated in two further small pilot studies. In a controlled study of 13 breast cancer patients, improvements in hematological toxicity were observed following a fasting period of 48 h [11]. In uncontrolled feasibility and dose-effect study, 20 patients under platinum chemotherapy were compared following 24-, 48-, and 72-h fasting periods, and reduced DNA damage was documented in patients fasting for 48 h or longer [10].

A previous pilot study prior to the trial described here, including 34 patients with either breast cancer (*n* = 30) or ovarian cancer (*n* = 4) undergoing chemotherapy in a crossover design, showed favorable effects of chemotherapy cycles with STF versus normocaloric chemotherapy cycles. STF during chemotherapy was safe and feasible, and positive effects on quality of life, well-being, and fatigue were observed [12].

We discuss whether the anti-cancer effects of fasting result from a reduction of insulin-like growth factor-1 (IGF-1), mTOR, and/or the regulation of p53 signal molecules that accompany it [21, 28, 29]. Since IGF-1 promotes cell proliferation and inhibits apoptosis, its reduction plays a key role in the protection against cancer in mammals [30, 31]. Experimental data suggest that reduced serum IGF-1 levels are not only a result of fasting, but can also be a result of dietary protein restriction [32]. Also, a short-term 50% caloric restriction in combination with a massive dietary protein reduction led to an improvement in chemotoxicity resistance in mice [32, 33]. A plant-based diet can be a very effective way of dietary protein restriction, as plants overall contain less protein than equal amounts of animal products and may therefore lead to a reduction of IGF-1 levels [34].

It thus remains an open research question whether beneficial effects of STF can be confirmed in larger clinical trials and whether they are due to the subtotal caloric restriction itself or to the absence of animal proteins and/or refined carbohydrates [35].

Based on the above, we designed a protocol for a multicenter randomized controlled clinical study that intends to test the following hypothesis. Chemotherapy for adjuvant and neoadjuvant treatment of breast cancer and ovarian cancer is more effective and better tolerated when combined with short-term fasting. We compare this to chemotherapy combined with a short-term plant-based diet, characterized by a restriction of animal protein and refined carbohydrates.

## Methods/design

### Study design

This is a multicenter, randomized controlled, two-arm intervention study with a parallel group assignment. The main objective of this study is to evaluate the effectiveness of the two above supportive dietary interventions in patients with breast cancer or ovarian cancer undergoing chemotherapy. Effectiveness is measured by the quality of life, reduction of chemotherapy-induced side effects, and histological remission in neoadjuvant regimes.

**Fig. 1:**
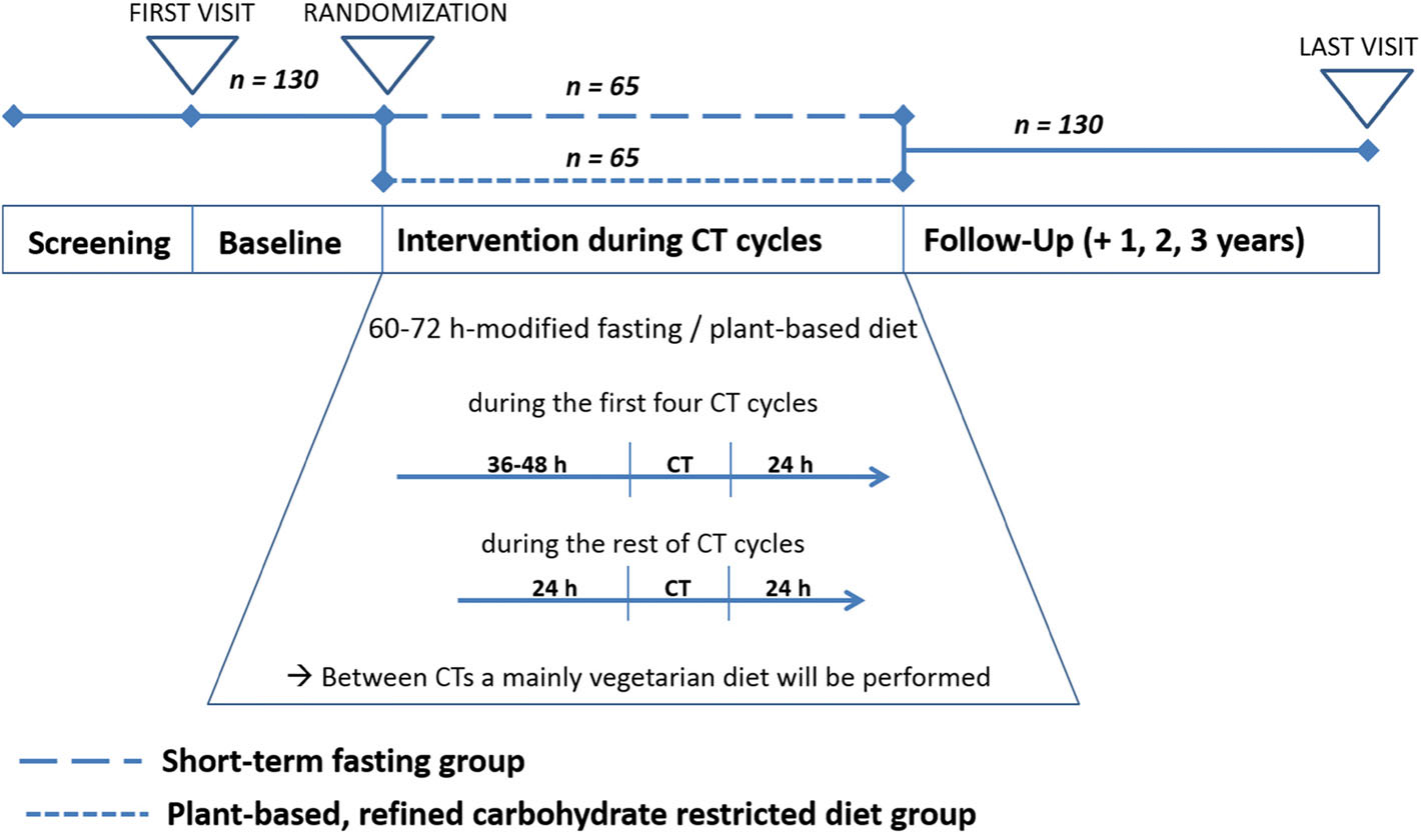
Study design

It is planned to include 150 female participants, including 120 patients with breast cancer and 30 patients with ovarian cancer. Recruitment started in May 2017 (ClinicalTrials.gov identifier NCT03162289). Patients are being recruited in 8 different specialized centers (7 hospital facilities and 1 outpatient center) for the treatment of gynecological cancers in three German cities (Berlin, Freiburg, Ludwigsburg) of two different German federal states. Study participants are being randomized to one of the two dietary interventions. All participants attend their respective study centers for six study visits: at baseline, after 4 months, at the end of their chemotherapy, and at follow-up visits 1, 2, and 3 years after baseline (see Fig. 1 for visualization).

The institutional review board of the Charité Universitätsmedizin Berlin as well as the ethics commissions of both the federal state of Baden-Württemberg and the Albert-Ludwigs-Universität of Freiburg approved the study protocol. All study participants have to submit written informed consent before they can be included in the research. The study will be conducted in accordance with the Declaration of Helsinki, as amended; the guidelines of the International Conference on Harmonization of Good Clinical Practice (ICH-GCP); and applicable German law. Monitoring will follow the ICH-GCP guidelines.

### Recruitment and randomization

For recruitment purposes, brochures with information about the study are being distributed in all participating study centers. Patients are invited to join the study by the investigators and the responsible study nurses, either directly when the diagnosis is communicated or as part of the tumor conference protocol. Detailed information on both background and protocol of the study is provided to the patients orally and in written form at least 24 h prior to the baseline visit, and all participants’ questions are answered before allowing them to sign the written informed consent form. On the consent form, participants are asked if they agree to the use of their data should they choose to withdraw from the trial. Participants are also asked to give permission to the research team to share relevant data with people from the cooperating research centers or from regulatory authorities. They are also informed that this trial involves collecting biological specimens for storage. The informed consent form can be found in Additional file 5 of this publication.

Eligibility of patients is determined during the screening visits by the investigators, through a screening document containing all inclusion and exclusion criteria. Only if all inclusion and no exclusion criteria are fulfilled can the patient be included in the study. Randomization is stratified by cancer type (breast or ovarian) and is based on block randomization with variable block length. Block randomization is a commonly used technique in clinical trial design to reduce bias and to achieve balance in the allocation of participants to treatment arms. This method increases the probability that each arm will contain an equal number of individuals by sequencing participant assignments by block. The random assignment of participants to one of the two intervention groups is carried out in a 1:1 ratio. The randomization procedure is performed by a web application designed for the study (https://natur.charite.de/randoFIT2). The application runs on a server of Charité Universitätsmedizin Berlin using Shiny Server [36]. Each study center has its own secure login to this web application.

The allocation sequence concealment secures strict implementation of the allocation sequence without foreknowledge of intervention assignments. First, the decision to accept or reject a participant will be made, then informed consent will be obtained from the participant, in ignorance of the next assignment in the sequence. After this, the allocation and the corresponding participant’s study ID are generated for this patient by the web application. These two values are transmitted from the Charité server to a tablet at the end of a questionnaire that the participant has to answer. The study personnel in direct contact with participants of the study, who carry out recruitment, visits, trainings, or phone calls, has no access to the randomization list. This personnel is not blinded, as there is direct contact with participants. Thus, the design is open-label with only the data analysts being blinded. The statisticians analyzing the data will be unblinded to the allocation, only after all the data have been collected, entered into the database, and cleaned. There are no interim analyses planned as we do not anticipate problems detrimental to the participants.

### Trial status

From May 2017 and presumably until August 2020, participants will be recruited. The third and currently the last protocol version has been translated and can be found in the supplements. This third amended protocol version was reviewed and approved by the ethics committee of Charité Universitätsmedizin Berlin in July 2018, followed by the ethics review boards of the Albert-Ludwigs-Universität in October 2018 and the medical association of Baden-Württemberg in December 2018.

### Participants

#### Inclusion criteria

The main inclusion criteria are a confirmed diagnosis of breast cancer or ovarian cancer without distant metastases, age between 18 and 75 years, and adjuvant or neoadjuvant chemotherapy with one of the following regimes:

- Breast cancer: 4× epirubicin or adriamycin (doxorubicin) and cyclophosphamide (EC or AC, respectively) tri-weekly, followed by 12 cycles paclitaxel weekly [37] or 4 cycles docetaxel tri-weekly [38]
- Ovarian cancer: chemotherapy according to current protocols, at least 4 planned cycles of chemotherapy containing carboplatin [39, 40]

#### Exclusion criteria

The exclusion criteria are existing underweight (BMI < 19 kg/m^2^) or weight reduction > 3 kg or > 5 kg in the last 1 or 3 months, respectively; a pre-diagnosed eating disorder (anorexia, bulimia, binge eating disorder); renal insufficiency (creatinine > 2 mg/dl); type 1 diabetes mellitus or insulin treatment; severe internal or psychiatric co-morbidity; terminal diseases; diseases with a significant reduction in mobility; inclusion in another study protocol; and absence of email and internet access.

Intervention discontinuation criteria are withdrawal of consent and medical reasons for stopping the intervention, for example, a reduction of BMI to < 18 kg/m^2^ or hospitalization due to chemotherapy-induced side effects. Participants discontinuing the intervention are asked to attend all remaining study visits and to fill out follow-up questionnaires outside the study protocol.

### Study interventions

The two study interventions are (1) STF and (2) a normocaloric plant-based diet with restriction of refined carbohydrates. In order to maximize the understanding of and the adherence to these two different dietary interventions, all participants receive a nutritional counseling session. Introductory nutritional counseling takes place in a time frame of 1 week to 2 days before the first chemotherapy, in a one-on-one, 60-min session with a specifically trained nutritionist. All nutritionists hold a diploma from one of the certified fasting academies of Germany [41, 42], where they have been trained in how to accompany fasters and counsel them on a plant-based wholefood diet. All participants will be called 1 to 2 days before and 1 day after the first four chemotherapy sessions by study nutritionists, for nutritional advice and support. At each phone call, the nutritionist also asks participants about their compliance with the dietary intervention through a semi-structured interview. Throughout the complete duration of the intervention, participants have the option of contacting the nutritionist via e-mail or mobile phone at any time. The nutritionist is able to contact the principal investigator and his medical team for any required medical advice from 08:00–22:00 every day including weekends.

#### Short-term fasting group

The method of STF, as used in our intervention, has its origins in a well-established approach of periodic (prolonged) fasting, which has been implemented in the Immanuel Hospital, Berlin, and other inpatient and outpatient settings in German-speaking countries (Germany, Austria, Switzerland) for several decades and is principally based on the Buchinger technique; this fasting approach has already been evaluated in clinical studies on rheumatic diseases and chronic pain conditions [43, 44].

Within the first four cycles of chemotherapy, participants follow a modified fasting regime of 60–72 h. The fasting period starts 36–48 h before each chemotherapy cycle (at 6 p.m. 2 days before receiving chemotherapy) and ends 24 h after each chemotherapy cycle. Participants are advised to follow a light plant-based diet before fasting, with a daily energy supply of about 500–700 kcal (on the second day before chemotherapy, from breakfast until 6 p.m. when they start to fast). During all fasting periods, participants are only allowed to consume vegetable juices and vegetable broths, with a maximum dietary energy intake of 350–400 kcal per fasting day. Unrestricted amounts of water and herbal teas are allowed. For more details on the exact information patients of the fasting group are given, including recipes and the timing of the intervention, please refer to Additional file 1.

In cases where participants take regular medication for other conditions, their medication might need to be adjusted during fasting days, as fasting is known to potentially reduce blood pressure [45], impact electrolyte balance, and influence blood sugar levels [46, 47]. It can also prolong bleeding time and may trigger attacks of migraine [44]. For this reason, diuretic, anti-hypertensive, and anti-diabetic drugs as well as coumarins will be adapted by physicians. Patients known to suffer from migraine headaches will be instructed to take their usual medication, as soon as the first signs of migraine appear during fasting. For more information on changes necessary in regular medication during STF, please refer to Additional file 2.

As Taxol treatments can be weekly, participants are advised not to follow the STF regime during these treatments. Instead, after having finished the first four chemotherapy cycles, all participants allocated to the fasting intervention are advised to follow only the light, plant-based diet described above, for two consecutive days at every Taxol application. This means that the day before and on the day of the Taxol application, participants are allowed a maximum caloric intake of 1000 kcal. In addition, they will be advised to fast for at least 14 h on the night preceding Taxol chemotherapy.

Between all chemotherapy appointments, the participants are encouraged to follow a wholefood lacto-vegetarian diet. To help in the implementation of this diet, a vegetarian cookbook is given to each participant, in addition to the handouts [48]. If participants indicate to the nutritionist that this is not feasible for them, they are advised to eat a maximum of two portions of meat or fish, and two eggs a week. Such deviations from the basic protocol are documented by the nutritionists during counseling. For more information regarding nutritional counseling on the recommendations given to participants on the vegetarian diet, please refer to Additional file 3.

During this period, participants allocated to the fasting intervention are additionally encouraged to follow a pattern of time-restricted eating, with 14 h of fasting overnight for at least 6 days per week.

#### Plant-based group

Participants allocated to this group are instructed to adhere to a normocaloric wholefood plant-based diet with restriction of sugar and refined carbohydrates. Within the first four chemotherapy cycles, patients maintain this plant-based diet for a duration of 60–72 h, starting 36–48 h before the beginning of chemotherapy and finishing 24 h after chemotherapy. After the first four chemotherapy cycles, during Taxol treatments, they should maintain 2 days of a plant-based diet with restriction of refined carbohydrates (24 h before and after chemotherapy). For more detailed information on this dietary intervention, please refer to Additional file 4.

For the rest of the intervention time, this group is encouraged to follow the wholefood lacto-vegetarian diet as described in the “Short-term fasting group” section, as well as in Additional file 3.

### Data monitoring

The data monitoring committee consists of a medical doctor, a study nurse, and an expert on bioinformatics. It closely monitors all study visit schedules from all cooperating centers and questionnaire reports from patients. All cooperating centers have the obligation to report any changes in study visit timings, or deviations from the chemotherapy protocol, to the monitoring committee. In case of any protocol violation, the committee will report to the study coordinator. The data monitoring committee is independent from the sponsor and free of competing interests. Further details about its operating procedures can be obtained by contacting the corresponding author via email.

Adverse events are documented at each visit, using specifically selected items of the National Cancer Institute Common Terminology Criteria for Adverse Events, as well as open questions. Adverse events are also recorded at any time between visits, when a patient reports such an event to the study personnel. Serious adverse events will be reported to the study coordinator and principal investigator as soon as they become known, in a time frame of 24 h. The principal investigator can, in close collaboration with the data monitoring committee and the study coordinator, take the decision to discontinue the study, either due to adverse or serious adverse events attributed to the study intervention or for the reasons mentioned in the “Exclusion criteria” section or because of failure to recruit enough patients. Interim analyses will not take place, as no detrimental health effects are to be expected. Internal audits will be undertaken by the study coordinator, including all participating study centers, every 6 months. All participating study centers have the obligation to regularly host external audits, which are undertaken by the certifying boards of the respective responsible medical associations [49].

#### Data dissemination policies

Trial results will be published in peer-reviewed journals, and participants will be invited to a public presentation of the results. On publishing the outcomes, the anonymized individual participant data that underlie the reported results, as well as the statistical code, will be made available to scientific investigators who issue a methodologically sound proposal. Nine months after publishing our results and at least for 3 years, the data will be deposited and available at the Charitè Universitiätsmedizin data warehouse without investigator support.

### Outcome measures

To assess the primary outcome (health-related QOL), we use the Functional Assessment of Chronic Illness Therapy (FACIT©) measurement system [50], the basis of which is the Functional Assessment of Cancer Therapy-General (FACT-G©). The FACIT© scales serve as a complement to the FACT-G© scale by covering relevant disease- or treatment-related issues which have not already been addressed in the general questionnaire (FACT-G©). The FACT-G© scale is assessed electronically at baseline and 2 days before and 7 days after each chemotherapy cycle in tri-weekly chemotherapies. Likewise, it is assessed 2 days before chemotherapy and once 7 days after the last cycle in weekly chemotherapies, and also at each study visit. For an overview of the visits, please refer to Table 1. The summarized change of the FACT-G© score will be analyzed.

**Table 1:** Visit overview

	Study period						
	Screening (visit − 1)	Baseline (visit 0)	Visit		Follow-up		
			1	2	3	4	5
**Week**	− 1	0	16 ± 2	3 (− 1/+ 2) weeks after Chemotherapy	48 ± 2	96 ± 2	144 ± 2
Enrolment							
Eligibility screen		X					
Informed consent	X						
Randomization		X					
Allocation		X					
Interventions^a^							
Intermittent fasting							
Plant-based, sugar-restricted diet							
Nutritional counseling		X					
Assessments							
Demographic data		X					
Medical history		X					
Concomitant medication		X	X	X	X	X	X
Anthropometric measurements		X	X	X	X	X	X
Vital signs (blood pressure, heart rate)		X	X	X	X	X	X
Bioelectrical impedance analysis		X	X	X	X	X	X
Physical examination		X	X	X	X	X	X
AE/SAE query			X	X	X	X	X
Blood panel Blood values for liver and renal function IGF-1, insulin, glucose, and ketone bodies in blood samples of subgroup^b^ IGF-1, insulin^b^ Ketone bodies^b^		X	Timing varies according to individual therapy plan.				
		X	Timing varies according to individual therapy plan.				
		X	X				
		Prior to the first chemotherapy prior to each of the first four chemotherapies					
Long-term explorative measurements: e.g., polyneuropathy, cardiomyopathy Frequency of recurrence					X	X	X
					X	X	X
CTCAE		X		X	X	X	X
Questionnaires							
FACT-G		X	X	X	X	X	X
TOI (= PWB+FWB+AC)		X	X	X	X	X	X
Total AC (FACT-B/FACT-O)		X	X	X	X	X	X
FACIT-F		X	X	X	X	X	X
FACT-Tax,FACT/GOG-Ntx		X	X	X	X	X	X
CIPNAT		X	X	X	X	X	X
HADS		X	X	X	X	X	X
Side effects of chemotherapy		X	X	X	X	X	X
Qualitative interviews in focus groups		X		X			

^a^Interventions take place only during chemotherapy

^b^Only in a subpopulation of *n* = 20

Secondary outcomes comprise complete remissions, determined by the number of histologically proven complete remissions (ypT0ypN0 or ypT0/is) in the surgical specimen after neoadjuvant chemotherapy. In this specimen, a classification according to the Miller and Payne score is also being carried out and the outcomes of both groups will be compared.

Further secondary outcome measures are the Trial Outcome Index, a measurement of physical aspects of QOL, which consists of the FACT-G© subscales of physical (PWB) and functional (FWB) well-being. For additional concerns, we used other tumor-specific (FACT-B© for breast cancer, FACT-O© for ovarian cancer) and therapy-specific (FACT Taxane© and FACT-F©) FACIT© scales. A modified version of the Chemotherapy-Induced Peripheral Neuropathy Assessment Tool (CIPNAT) [51], Hospital Anxiety and Depression Scale (HADS), and elective items of the Common Terminology Criteria for Adverse Events (CTCAE) [52] are further secondary outcome measures.

Furthermore, complete blood count (CBC) without differential is documented at baseline and at nadir (day 7, 8, 10, or 11) (standard documentation according to the guidelines of the Association of German Tumor Centers). At all visits, adverse events, anthropometric measurements, blood pressure, and heart rate are recorded. At each visit, patients are also asked to complete an electronic questionnaire about their eating behavior, side effects of chemotherapy, and quality of life. Blood values for liver (GPT, GOT, GGT, AP) and for renal (creatinine, urea) function are measured in a center-specific manner.

In a subgroup (*n* = 20), explorative measurements of IGF-1, insulin, and blood glucose will be carried out in blood samples at baseline, the day of the first chemotherapy, and at V1 (4 months after baseline). Furthermore, in order to assess patient compliance, ketone bodies are measured in fingertip blood samples at baseline and V1 as well as during the first four cycles of chemotherapy just before administering the chemotherapy. These blood samples are collected by a trained nurse or physician, analyzed by our university laboratory as routine blood parameters, and stored for a few days. The specimens are not stored for any further use.

In the subpopulation recruited in the Freiburg center (anticipated *n* = 10), a non-invasive measurement of body composition is performed during the first four chemotherapy cycles on the day of chemotherapy. These measurements are carried out using bioelectrical impedance analysis.

For long-term explorative measurements, such as the frequency of tumor recurrence or incidence of polyneuropathy and cardiomyopathy, information is taken from the patient files, visits, and questionnaires.

Compliance with STF and the plant-based diet regimen is assessed by telephone calls around the time of each of the first four chemotherapy appointments. Additionally, we assess compliance in the electronic questionnaires that are periodically filled in, before and after chemotherapies, as well as 3 months after the end of the chemotherapy, through a separate electronic questionnaire developed by our group. The translated questionnaires can be found in Additional file 6.

In addition, we carried out a qualitative assessment in the form of 45-min focus-group interviews [53] in which the acceptance of the respective nutritional intervention, as well as the implementation of the intervention, is evaluated after chemotherapy with 12 volunteers (6 from the fasting and 6 from the plant-based group).

For a detailed overview of time points for questionnaires, see Table 2.

**Table 2:** Questionnaire overview

	**Treatment according to Henderson scheme**														
	1. AC/EC q3		2. AC/EC q3		3. AC/EC q3		4. AC/EC q3		1. Taxan q3		2. Taxan q3		3. Taxan q3		4. Taxan q3
**Days before/after chemotherapy**	− 3 days (only if V0 > 7 days before)	+ 7 days	− 3 days	+ 7 days	− 3days	+ 7 days	− 3 days	+ 7days	− 3 days	+ 7 days	− 3 days	+ 7 days	− 3 days	+ 7 days	− 3 days
	**Treatment according to Sparano scheme**														
	1. AC/EC q3		2. AC/EC q3		3. AC/ECq3		4. AC/ECq3		1. Taxan q1		2. Taxan q1		3.-11. Taxan q1		12. Taxan q1
**Days before/after chemotherapy**	− 3 days (only if V0 > 7 days before)	+ 7 days	− 3 days	+ 7 days	− 3 days	+ 7 days	− 3 days	+ 7 days	− 3 days		− 3 days		− 3 days		− 3 days

### Data analysis (sample size and statistical analysis)

The sample size was calculated using a 2-sided Wilcoxon-Mann-Whitney test. The sample size with a fasting group of 67 patients and a plant-based group of 67 patients reaches a power of 80% at a significance level of *p* = 0.05 to detect an effect size of 0.5 for FACT-G© between these two groups. If an additional 10% dropout rate is assumed, we need a minimum of 148 patients. With a ratio of 4:1 between breast cancer and ovarian cancer patients, we need a total of 150 patients (120 breast cancer and 30 ovarian cancer patients), divided into 75 patients each (60 breast cancer and 15 ovarian cancer patients) for the STF group and the plant-based group. All FACIT© scales are designed with a higher score indicating better well-being. Accordingly, we reversed response scores on negatively phrased questions. The scores will be obtained in accordance with the formula that was previously established by the FACIT© system. In cases where individual questions are skipped, scores will be prorated using the average of the other answers in the subscale (prorated subscale score = [sum of the item scores] × [*N* of items in subscale]/[*N* of items answered]) as long as more than 50% of the items are answered (minimum of 4 items for the subscales). The FACT-G score is considered appropriate as long as at least 22 of 27 FACT-G items are completed (≥ 80%). Inter-subscale correlations will be computed using Pearson correlation, and the reliability of the internal consistency for all scales will be assessed by computing Cronbach’s alpha. When Cronbach’s alpha exceeded 0.90, the scale is considered to have sufficient precision for individual classification or diagnosis. The minimal important differences (MIDs) of STF and plant-based groups, i.e., the “smallest change in the score that patients perceive as important, either beneficial or harmful, and that would lead the clinician to consider a change in the patient’s management” will be used to find clinically meaningful improvements [54]. MID values over 3–7 (mean 5) for FACT-G© and over 3–4 for the fatigue subscale and 6 for total FACT-F will be considered significant. Normality will be tested with the Shapiro-Wilks test. Furthermore, various methods for adjustment at the statistical analysis stage will be applied. We will use “change score analysis” that determines group effect, based on the difference between the baseline and the post-treatment score (basic adjustment) and analysis of covariance. This is a model-based adjustment that includes the baseline of the outcome variable in the model. Statistical adjustment can also be performed by the use of logistic regression or by pooling the stratified analyses, using, for example, a Mantel-Haenszel test. We will simultaneously use these various design methods that reduce covariate imbalance and statistical adjustment during analysis. Statistical analysis will be performed using R version 3.5.1 (R Foundation) and IBM SPSS Statistics, version 26.0 (IBM Corp., Armonk, NY, USA).

## Discussion

This is the first randomized controlled clinical trial evaluating the effects of intermittent short-term fasting during chemotherapy on QOL and, in a subpopulation, on histological tumor remission. It is also the first trial to compare the effects of intermittent STF with an intermittent short-term plant-based diet in such a setting.

### Strengths

One of the main strengths of this study is its large sample size, uncharacteristic for an innovative and unusual dietary intervention approach in oncology. To our knowledge, this is the largest confirmatory study on dietary interventions involving fasting in the context of chemotherapy. Also, the randomized controlled study design adds to this strength. The fact that baseline questionnaires are filled out by the patients before the interventional allocation might compensate for placebo effects at baseline. Another strength of this study is that it focuses on both histological data and multiple patient-related outcomes such as fatigue and quality of life. This will provide a deep insight into the physical and psychological effects of both dietary interventions on the patients. Furthermore, as a result of close electronic outcome monitoring, we expect fairly complete data sets. Also, in addition to routine laboratory measurements, we are investigating exploratory molecular markers such as IGF-1 in a subpopulation. These data will allow us to further explore the mechanisms of intermittent STF and the effects of an intermittent short-term plant-based diet accompanying chemotherapy, to create a basis for further treatment options. Finally, this is the first intervention study that examines whether a beneficial health effect results only from fasting, or whether it might also be achieved through a plant-based diet with restriction of refined carbohydrate intake.

### Limitations

A dietary intervention as an adjunct to chemotherapy is an easy and cost-effective way to improve the required treatment, if successful. However, carrying out a controlled study on a dietary intervention has known challenges, as blinding and placebo control are not possible and thus non-specific effects cannot be ruled out. Furthermore, adherence to a dietary intervention is difficult to assess objectively. This difficulty is specifically present in our study, because of the outpatient setting and the relatively long study duration. Even though we track dietary adherence through different methods (questionnaires, phone calls by nutritionists, study visits), and all study personnel is trained to encourage patients to honestly disclose all food and beverages consumed during the STF or the plant-based diet periods, this cannot rule out the possibility of under- or misreporting.

As fasting cures enjoy a high popularity in Germany, a waiting-list control group undergoing no dietary modification was unlikely to work. Still, disappointment with the interventional dietary allocation could cause some patients allocated to the control group to follow the STF intervention without informing the study personnel. In our pilot study [12], some of the participants that had been allocated to the STF group first did not discontinue fasting when they were, due to the crossover design, asked to stop STF. They argued that they felt STF improved their health status in such a way that they no longer wanted to adhere to the study protocol. To prevent such protocol violations due to individual preferences, we designed this study with a putatively effective dietary control intervention as a comparator [33]. As shown in the introductory section of this publication, a combination of mild caloric restriction with a strong protein restriction had some fasting-mimicking effects in mice [35, 55]. For the design of this study, we postulated that a mild protein restriction might also enhance the conventional treatment and lower side effects, but not to the same extent as STF. The selected control intervention may reduce and dilute between-group differences, if the effect of protein and sugar restriction approximates the “true” STF effect. So, for feasible reasons, reducing drop-outs after randomization and enhancing participant compliance, we designed the control intervention as described, accepting the risk that the effects of STF itself might not be detectable anymore when the groups are compared. If this should be the case, we will consider comparing the results of our cohort with outcomes of conventionally treated, similar cohorts published elsewhere.

A further limitation of this study is the multicenter setting. Of course, it is also a major strength, since center-specific effects can be diluted and more general conclusions drawn. But although a certification process for specialized breast cancer centers in Germany exists [49], procedures in clinical reality can vary substantially. Some of the participating centers undertake only the surgical part of the treatment and outsource chemotherapies to different outpatient centers. Other centers perform all relevant procedures themselves, from surgery to chemotherapy and follow-up interventions including radiotherapy. This creates a diversity of settings, in which chemotherapy is applied. It is a challenge to establish standard operating procedures in each of these settings.

One of the problems of adapting existing procedures to our requirements can be exemplified by the co-therapeutic applications of cortisone infusions. Theoretically, cortisone infusions would counteract the effect of both dietary interventions, because cortisone acts directly on the IGF-1 pathway. But in all cooperating centers, cortisone infusions belong to standard care and cannot be adapted for the purpose of this study for practical reasons.

The preclinical data and existing clinical trials concerning short-term fasting and protein restriction are promising. Thus, the FIT2 study has the potential to provide essential data on the effects and efficacy of intermittent short-term fasting and an intermittent short-term plant-based diet in patients with ovarian or mamma carcinoma during chemotherapy. Taken together, this approach will help to provide clearer and more knowledgeable predictions on the safety and effectiveness of such dietary interventions accompanying conventional approaches.

### Recruitment status

This study is currently recruiting patients.

## Supplementary information

**Additional file 1.** Handouts for fasting group.

**Additional file 2.** Standard operating procedures for adapting medication during fasting.

**Additional file 3.** Handout on vegetarian wholefood diet.

**Additional file 4.** Handouts for normocaloric plant-based wholefood diet.

**Additional file 5.** Informed consent form.

**Additional file 6.** Translated questionnaires.

## Data Availability

All relevant data is available in the supplements. For any further inquiries, please contact the corresponding author.

## Abbreviations

Akt: Protein kinase B
CIPNAT: Chemotherapy-Induced Peripheral Neuropathy Assessment Tool
CTCAE: Common Terminology Criteria for Adverse Events
DSR: Differential stress resistance
FACT Taxane: Functional Assessment of Cancer Therapy-Taxane
FACT/FACIT: Functional Assessment of Chronic Illness Therapy
FACT-B: Functional Assessment of Cancer Therapy-Breast Cancer
FACT-F: Functional Assessment of Cancer Therapy-Fatigue
FACT-G: Functional Assessment of Cancer Therapy-General
FACT-O: Functional Assessment of Cancer Therapy-Ovarian Cancer
FWB: Functional well-being
HADS: Hospital Anxiety and Depression Scale
ICH-GCP: International Conference on Harmonization of Good Clinical Practice
IGF-1: Insulin-like growth factor 1
mTOR: Mammalian target of rapamycin
PWB: Physical well-being
QOL: Quality of life
ROS: Reactive oxygen species
STF: Short-term fasting
