# Supplementary material for: Short-term fasting accompanying chemotherapy as a supportive therapy in gynecological cancer: protocol for a multicenter randomized controlled clinical trial
Source: Trials. 2020 Oct 15;21:854. doi: 10.1186/s13063-020-04700-9 (PMC7559781; doi:10.1186/s13063-020-04700-9)
Supplement: Supplementary file 1 — Additional file 1. Handouts for fasting group. [file 13063_2020_4700_MOESM1_ESM.pdf]

Protocol for fasting accompanying chemotherapy (CT)

<u>1st - 4th chemotherapy</u>

A day with a light plant-based diet, in which protein, fat and fast absorbable carbohydrates are not recommended (see meal plan), is to be performed before the start of the fasting period and at its end.

Fasting starts at the evening of the day with the light plant-based diet at 6 p.m. and ends 24h after the end of the drug infusions.

Day 1		Day 2	Day 3	Day 4		Day 5
Start: 2 days before CT		1 day before CT	day of CT	1 day after CT		End
Light plant-based diet	6 p.m. start of fasting	fasting	fasting	fasting until 24h after end of CT	Light plant-based diet*	Light plant-based diet*

*until 24 h after end of fasting

<u>From 5th chemotherapy onwards</u>

Here we recommend a light plant-based diet on the day before and the day of chemotherapy. In between the two days a 14 h intermittent fasting is to be performed, meaning not to eat anything from 6.p.m. the day before chemotherapy until 8 a.m. of the chemotherapy day, while drinking plenty of calorie-free beverages.

Day 1		Day 2	
Day before CT		Day of CT	
Light plant-based diet	6.p.m. start of fasting	8 a.m. end of fasting	Light plant-based diet

<u>In between chemotherapies</u>

In between chemotherapies we recommend you to follow a vegetarian wholefood diet (see separate recommendation sheet) in combination with a 14 h intermittent fasting regime. This could be as following: you eat until 6 p.m., then you stop eating while consuming plenty of calorie-free beverages, and you can start eating again at 8 a.m. Or you can just skip one meal (breakfast or dinner). We recommend you to follow the 14 h intermittent fasting regime on at least six days a week.

Light plant-based diet

The light plant-based diet day facilitates circulation and digestion and prepares the body for the fast. You should for this purpose reduce your diet to certain foods (organic vegetables are very suitable), prepared without additional fat, proteins or sweeteners. On such a day you should try to consume at least two litres of mineral water or unsweetened herbal tea.

Here you will find some suggestions of for your meals during such a light plant-based diet day.

Examples of a meal plan for a light plant-based diet

Rice day

Three times daily 50 g whole grain basmati rice, 50 g millet or 50 g quinoa cooked in 100 ml water until soft. In the morning served with 200 g berries or 150 g freshly grated unpeeled apple, for lunch and dinner with 200 g steamed tomatoes or other vegetables, fresh herbs (parsley or basil) and if needed a little salt for the taste.

Potato day

600-700g low-starch cooked potatoes to be distributed to 3 meals. The potatoes should be prepared as potatoes in the skin or oven potatoes (e.g. with fresh herbs such as marjoram, parsley, thyme, chives, dill or caraway). These are served with approximately 200 g vegetables per meal, e.g. for breakfast with fresh tomatoes topped with chives, lemon and curry powder, for lunch and dinner with steamed vegetables and fresh herbs. If needed, add a little salt.

Oat day

Three times daily 50 g rolled wholegrain oats, soaked shortly in water and served with 200 g of berries or 150 g freshly grated unpeeled apple with cinnamon and pure vanilla powder. Alternatively serve with 200 g of steamed vegetables and fresh herbs. Add some salt, if needed.

Vegetable day

Breakfast: Mix 200 g of grated carrots and one medium size grated apple with some lemon juice, cinnamon or pure vanilla powder (optionally add 1 tsp olive- or linseed oil)
Lunch: e.g. 200 g steamed cauliflower and 100 g steamed broccoli or 200 g steamed pumpkin and 100 g steamed kohlrabi with one medium size low-starch potato. If well tolerated a salad can be prepared alternatively with 50g leafy greens (e.g. chicoree, iceberg or lamb's lettuce), 1 medium size tomato, 6 radishes, one quater cucumber with fresh herbs, lemon juice, white pepper, a little bit of sea salt and if needed 1 tsp olive- or linseed oil.
Alternatively you can prepare a salad instead of steamed vegetables. This could consist of 50 g leaf lettuce (e.g. chicory, iceberg lettuce, mache), 1 medium size tomato, 6 small radishes, a quarter of a green cucumber with lemon juice, fresh herbs, white pepper and a little salt. If necessary, add a tsp of olive- or linseed oil.
Dinner: steamed vegetables as recommended for lunch

Recipes for different fasting broths

Recipes are for 4 portions

Potato-Broth

1 liter of water
250 g potatoes
250 g carrots
1 leek
1 large parsley root
1/2 tsp caraway seeds
optionally parsley, sea salt, granulated vegetable broth

Wash and cut the vegetables into pieces and bring them to boil in a pot with the water. Let it simmer for 30 to 45 minutes. Add the caraway after 15 minutes. Filter the soup so that no pieces remain in the broth. Serve the broth with some fresh parsley and, if needed, add some salt or granulated vegetable broth. Other possible herbs are: lovage, oregano, dill, basil, nutmeg, marjoram.

Basic vegetable broth

1liter of water
1 kg vegetables (e.g. carrots, tomatoes, pumpkin, broccoli, zucchini)
1/2 bay leaf
1 clove
1 juniper berry
1-2 tbsp of fresh herbs (e.g.parsley, oregano, marjoram, basil, dill, lovage)
Optionally a little bit of salt

Wash the vegetables and cut them into pieces. Put them in a pot with water and bring it to boil. Let it simmer for 30 to 45 minutes. After 15 minutes add the bay leaf, clove and juniper berry. Filter the soup to get the broth. Serve the broth with fresh herbs and if needed add some salt.

Variation: Tomato broth
Use fresh tomatoes for your vegetable broth and add 1-2 tsp of tomato purée. Serve with fresh basil.

Additional tipp: tomato broth: add fresh tomatoes and 1-2 tbsp tomato paste and fresh basil.

Liver compress

A rest at noon with a liver compress can help get through a fasting day easier.

<u>Things needed for a liver compress</u>

2 hot water bottles

1 kitchen towel

1 towel

1 bowl

1 blanket

<u>Instructions</u>

1. First of all, fill the hot water bottles with hot (not boiling!) water
2. Check if they are properly closed
3. Put the kitchen towel in a bowl with hot water and wring it out
4. Fold the dish cloth twice or trice and place it on the right upper abdomen under the costal arch and lay a hot water bottle on top.
5. Fix the hot water bottle with the towel or a tight t-shirt.
6. The second hot water bottle can be used to warm the feet.
7. Cover up with the blanket so that you feel comfortably warm.
8. Stay in this position for 30 minutes and enjoy the rest.
