# Supplementary material for: Short-term fasting accompanying chemotherapy as a supportive therapy in gynecological cancer: protocol for a multicenter randomized controlled clinical trial
Source: Trials. 2020 Oct 15;21:854. doi: 10.1186/s13063-020-04700-9 (PMC7559781; doi:10.1186/s13063-020-04700-9)
Supplement: Supplementary file 2 — Additional file 2. Standard operating procedures for adapting medication during fasting. [file 13063_2020_4700_MOESM2_ESM.pdf]

In case a patient takes medication and is randomized to the **fasting group**, the following medication should be adapted:

1. **Antihypertensives**
   1.1. **ACE inhibitors and Sartans:** Half dose during fasting days. If halving is not possible due to low dose, pause this medication during fasting days.
   1.2. **Diuretics:** Due to shifts in fluid and electrolyte balance while fasting, pause completely during fasting.
   1.3. **Combination preparations (with diuretic)**: Since the diuretics must be paused, please give the patient the additional individual substances in half dosage for the fasting days.
   1.4. Do not pause **b-blockers**!

   If the patients have their own blood pressure monitor, encourage patients to self-monitor their blood pressure at home.

2. **Oral antidiabetics**:

   Oral antidiabetics are to be stopped from the 1st day of fasting until the end of fasting (applies to **biguanide/metformin, sulfonylureas, glinides, glitazones, α-glucosidase inhibitors, DPP-4 inhibitors, SGLT-2 inhibitors**). From the first day of fasting, patients should measure their blood glucose daily for three days and seek medical advice if their blood glucose exceeds 12 mmol/l (216 mg/dl). Please communicate clearly to the patient that temporarily high blood glucose levels of up to 12 mmol/l are harmless, while hypoglycaemias that can occur if they take their medication during fasting can be life threatening!

3. **Anticoagulants**
   3.1. **Marcumar:** Half dose on the third day of fasting, then return to normal dosage.
   3.2. **NOACs:** No changes in medication necessary during fasting.

4. **Procedure for patients known to suffer from migraines:**
   Inform the patient that migraine attacks during fasting are likely. The patient should therefore take her usual prophylaxis (e.g. triptans) as early as possible at the onset of the symptoms!
