# Supplementary material for: Short-term fasting accompanying chemotherapy as a supportive therapy in gynecological cancer: protocol for a multicenter randomized controlled clinical trial
Source: Trials. 2020 Oct 15;21:854. doi: 10.1186/s13063-020-04700-9 (PMC7559781; doi:10.1186/s13063-020-04700-9)
Supplement: Supplementary file 3 — Additional file 3. Handout on vegetarian wholefood diet. [file 13063_2020_4700_MOESM3_ESM.pdf]

Vegetarian wholefood diet

The vegetarian wholefood diet is needs-based and contains all essential nutrients.

General recommendations:

- All foods should be:
  - as natural und unprocessed as possible
  - gently prepared/cooked
  - seasonal
  - regional
  - without artificial additives and perservatives
- No refined sugar or artificial sweeteners

Daily recommendations:

- At least 3 portions of vegetables (cooked or raw) and 2 portions of fruits (berries, oranges, apples)
- Several portions of wholegrain products (like wholegrain bread, rice, pasta, oats, millet, quinoa, amaranth)
- 30-40 g oil and fat (cold-pressed linseed, olive and rape seed oil, butter)
- 30 g-50 g nuts
- 1 fat-reduced fermented dairy product (e.g. 300 ml yoghurt, soured milk with 1,5% fat content or 50 g of fat-reduced hard cheese)
- 1.5-2 litres calorie-free beverages (water, unsweetened herbal tea)

Weekly recommendations

- Potatoes, legumes, tofu and other soy-products
- No refined sugar or artificial sweeteners should be used. If sweetening seems necessary, a small amount of honey, maple-, apple-, pear- or agave syrup can be used

Restricted foods

- Fish or meat should not be consumed more than twice weekly
- Eggs and other animal products apart from the above mentioned dairy products should not be consumed daily
