# Supplementary material for: Short-term fasting accompanying chemotherapy as a supportive therapy in gynecological cancer: protocol for a multicenter randomized controlled clinical trial
Source: Trials. 2020 Oct 15;21:854. doi: 10.1186/s13063-020-04700-9 (PMC7559781; doi:10.1186/s13063-020-04700-9)
Supplement: Supplementary file 4 — Additional file 4. Handouts for normocaloric plant-based wholefood diet. [file 13063_2020_4700_MOESM4_ESM.pdf]

Protocol for plant-based wholefood diet with restriction of refined carbohydrates accompanying chemotherapy (CT)

<u>1st - 4th chemotherapy</u>

The dietary intervention is to be performed from 6 p.m. two days before chemotherapy until 24h after the end of the drug infusions. We recommend you not to eat any animal products including meat, fish, milk, dairy products and eggs as well as to use less sweeteners. You are allowed to drink plenty of calorie-free beverages like mineral water and unsweetened teas.

Day -2	Day-1	Day 0	Day 1
2 days before CT	1 day before CT	day of CT	1 day after CT
6 p.m. start of plant-based diet (no animal products and less sweeteners) until 24h after end of CT			

<u>From 5th chemotherapy onwards</u>

We recommend to follow a plant-based diet the day before chemotherapy and during the day of the chemotherapy.

Day -1	Day 0
day before CT	day of CT
plant-based diet	

<u>In between chemotherapies</u>

A vegetarian wholefood diet is recommended in between chemotherapies (see separate recommendation sheet)

Recommendations for plant-based diet accompanying chemotherapy

General recommendations:

- Avoid all animal products including meat, fish, milk, dairy products, eggs and honey
- Restrict refined carbohydrates
- No fast absorbing carbohydrates
- The foods should be:
  - as natural und unprocessed as possible
  - gently prepared/cooked
  - seasonal
  - regional
  - without artificial additives and perservatives

Specific recommendations:

- At least 3 portions of vegetables (cooked or raw) and 2 portions of fruits (berries, oranges, apples) a day
- Several portions of wholegrain products (like wholegrain bread, rice, pasta, oats, millet, quinoa, amaranth)
- Plenty of oil and vegetable fats (cold-pressed linseed, olive and rapeseed oil, rich in omgea-3-fatty acids)
- 50 g nuts or nutbutter (walnut, almond, brazil nut, hazelnut, sesame)
- 250 ml of calcium enriched rice- or oat-drink
- 1.5-2 liters of calorie-free beverages (water, unsweetened herbal teas)
- You may complement your dishes with some potatoes, legumes, tofu and other soy-products
- No refined sugar or artificial sweeteners should be used. If sweetening seems necessary, a small amount of maple syrup can be used

Meal plan for plant-based diet

Breakfast

- Porrdige with oats, other rolled whole grains or millet
    - Recipe: Cook rolled grain or millet in water or plant based drink (e.g. rice- oder oat-drink) until soft. Add fruits like berries, grated apple, orange or mandarin slices, spices (e.g.cinnamon) and a little bit of maple syrup if required.

- Wholegrain bread with vegetable magarine rich in omega-3-fatty acids, vegan spreads, tomato, radish, fresh herbs or in a sweet variation with nutbutter (e.g. 100% cashew or almond butter)

Lunch

- Potatoes, wholegrain rice or pasta, millet, quinoa
    - with ample vegetables (any except corn)
    - prepared with rapeseed or olive oil
    - you may add tofu or legumes to the dish, e.g. in form of a patty
- Side salad: leaf lettuce, tomato, cucumber, radish, onions, garlic etc. Add herbs, vinegar, oil and if you like also mustard, spices, pepper, salt etc.
- Desert: one piece of fresh fruit (see breakfast selection) or nuts

Dinner

- Vegetable soup
- Wholegrain bread with margarine or vegan spreads, tomato, avocado, radish or salad (see lunch)

Beverages

- 1.5-2 liters calorie-free drinks (e.g. water, unsweetened herbal tea)
