# Supplementary material for: Short-term fasting accompanying chemotherapy as a supportive therapy in gynecological cancer: protocol for a multicenter randomized controlled clinical trial
Source: Trials. 2020 Oct 15;21:854. doi: 10.1186/s13063-020-04700-9 (PMC7559781; doi:10.1186/s13063-020-04700-9)
Supplement: Supplementary file 5 — Additional file 5. Informed consent form. [file 13063_2020_4700_MOESM5_ESM.pdf]

University outpatient clinic of Complementary and Alternative Medicine
Charité Universitätsmedizin Berlin
Königstrasse 63, 14109 Berlin; Tel. 030-80505-691/ FAX 030-80505-692
Email: naturheilkunde@immanuel.de

Declaration of consent

for participation in the scientific study on

Repeated Short-term Fasting accompanying chemotherapy as a Supportive Therapy in Gynecological Cancer: Protocol for a Multicenter Randomized Controlled Clinical Trial

Patient: ____
(surname, first name)

Date of birth: __ __. __ __ . __ __   Pat.-No.________________

The information about the clinical study was given to the patient by:

____
(Name of the doctor providing information in capitals)

and covered the following points:

- Type and objective of the clinical trial.
- Type and implementation of study interventions, study examinations with questionnaires and clinical examinations.
- Right to withdraw from the clinical trial.
- Data protection: Documentation, passing on and publication of patient data takes place in pseudonymized form, right to deletion of personal data and responsible authorities for right of appeal.
- A copy of the patient information form and the signed consent form was given to me.

With her signature the patient declares:

I wish to participate in the dietary intervention with random allocation to one of two intervention groups: Fasting or vegan, low-glycemic diet.

I agree to participate in this study and certify that the informative discussion covered the above points. I was informed verbally and in writing about the nature, significance, scope and risks of the scientific investigation in the context of the above-mentioned study and had sufficient opportunity to clarify my questions in an interview with the study physician. In

particular, I have understood the patient information presented to me and have received a copy of it and this declaration of consent.

I also agree that my contact data such as name, telephone number, email address and private address as well as chemotherapy regimen and treatment practice may be passed on to the responsible colleagues at the study center (see below) in order to enable regular surveys to be carried out as part of the mailing and follow-up of the questionnaires. I may be contacted either by telephone, SMS or email, should appointments or reminders regarding the questionnaires be necessary.

I understand that I may revoke my consent to participate in this clinical trial at any time without having to give reasons and that this will not adversely affect my further treatment. All my questions about the trial have been answered.

Privacy policy

The processing of your personal data takes place on the basis of the basic data protection regulation of the EU and the Berlin Data Protection Act.

The study physician is the data processor within the meaning of the EU Data Protection Regulation. By signing the declaration of consent, you agree that the study physician and his or her colleagues may collect and use your personal data for the purpose of the above-mentioned study for the conduct of the study and for research purposes in the field of integrative oncology. Personal data is, for example, your name, date of birth, your address and data on your health or illness or other personal data that was collected during your participation in the study or in one of the follow-up examinations for a specific purpose.

Furthermore, you agree that relevant information about your state of health generated during the medical treatment of the cancer (e.g. blood values or information about the course of chemotherapy) may also be used for the evaluation of the study results and passed on by the treating practices to the study supervisor (see below).

Your personal and health-related data will be stored exclusively on the in-house servers of your treatment center or servers of the Charité or Immanuel Diakonie. Health-related data is stored and processed exclusively under a pseudonym (meaning it is provided with a code that replaces your name).

The study physician gives personal and medical data, which have been generated in relation to the study, as well as the data of your treatment, to the study supervisor Prof. Dr. Andreas Michalsen (see address below) and his working group for the central administration of the data, for cases of adverse events, for data monitoring, for contacting for the necessary purposes of the study and for statistical analysis.
The study supervisor provides pseudonymized study-related data collected during the study for statistical evaluation to:

Prof. Dr. Manfred Wischnewsky
Department of Mathematics and Computer Science
University of Bremen
Universitätsallee 10-12
28359 Bremen
Tel.:+4942121861400
wischnewsky@escience.uni-bremen.de

The data available at the aforementioned locations will be stored for a period of 10 years and then destroyed.

You have the right to access (including a free copy) all personal data about you that is held by the study physician or the study sponsor. You also have the right to have incorrect personal data corrected. Furthermore, you have the right to revoke your consent to data processing at any time; in the event of such a withdrawal, you can demand the deletion of your personal data. The health and study related data would in this case be used anonymously (no longer related to your person) for statistical evaluation. To exercise these rights, please contact your study physician. You will find the address and telephone number at the end of this form.

Please note that the results of the study may be published in medical literature, but your identity will remain anonymous.

You may at any time object to the further processing of your data collected within the scope of the above-mentioned study and/or further examination of the samples taken from you and demand their deletion or destruction.

You also have the right to appeal to the supervisory authority responsible for data protection.

If you have any questions regarding data processing and compliance with data protection requirements, please contact the Charité Data Protection Office:
Stabsstelle Datenschutz
Charitéplatz 1
10117 Berlin
Tel. +4930450580016
E-Mail: datenschutz@charite.de

You have the right to file a complaint with the regulatory authority if you believe that your study data is being used in violation of applicable data protection laws.

For Berlin, this is the following regulatory authority:
Berliner Beauftragte für Datenschutz und Informationsfreiheit
Friedrichstraße 219
Tel.: +493013889-0
Fax: +49302155050
E-Mail: mailbox@datenschutz-berlin.de

Study supervisor:

Prof. Dr. med. Andreas Michalsen
Stiftungsprofessur für klinische Naturheilkunde
Charité Hochschulambulanz für Naturheilkunde
am Immanuel Krankenhaus Berlin
Königstrasse 63, 14109 Berlin
Tel. 030-80505 691
Fax. 030-80505 692
Email: a.michalsen@immanuel.de

Responsible study physician:

Daniela Liebscher
Prüfärztin
Charité Hochschulambulanz für Naturheilkunde
am Immanuel Krankenhaus Berlin
Königstrasse 63, 14109 Berlin
Tel: 030-80505 770
d.lieberscher@immanuel.de

Patient's consent to study participation and data processing:

____
Place, Date

____
Patient's signature

Declaration of the study physician:

Hereby I declare that on ______________________

I have informed the above-mentioned participant orally and in writing about the nature, significance, scope and risks of the above-mentioned study and that I have provided her with a copy of the information and this declaration of consent.

____
Place, Date

____
Signature of the doctor informing the patient
