# Supplementary material for: Short-term fasting accompanying chemotherapy as a supportive therapy in gynecological cancer: protocol for a multicenter randomized controlled clinical trial
Source: Trials. 2020 Oct 15;21:854. doi: 10.1186/s13063-020-04700-9 (PMC7559781; doi:10.1186/s13063-020-04700-9)
Supplement: Supplementary file 6 — Additional file 6. Translated questionnaires. [file 13063_2020_4700_MOESM6_ESM.pdf]

Compliance question

This question is part of the electronic questionnaires that are periodically filled out during the chemotherapy.

To what extent were you able to adhere to the dietary intervention?

**0 %**	**25 %**	**50 %**	**75 %**	**100 %**

The following questionnaire is developed by us and sent electronically to the relevant intervention group 3 months after the last chemotherapy.

Questionnaire for dietary intervention fasting accompanying chemotherapy

Questions concerning nutrition Part 1

1) How did you like the fasting rations?

Not at all	Little	Neither liked nor disliked	Fairly	Very much
1	2	3	4	5

2) How difficult or easy was the preparation of the fasting rations for you?

Difficult	Rather difficult	Neither difficult nor easy	Rather easy	Easy
1	2	3	4	5

3) Did it require you to make an effort to maintain the fast?

No	Little	Average	Pretty much	Very much
1	2	3	4	5

4) Could you imagine fasting again in the future?

No	Rather no	Maybe	Very likely	In any case
1	2	3	4	5

A <u>vegetarian wholefood diet</u> means the consumption of vegetable foods and low-fat sour milk products (e.g. yoghurt and soured milk with 1.5% fat or low-fat hard cheese) and the simultaneous avoidance of meat, fish, eggs and other dairy products.

5) In comparison to your previous diet before the chemotherapy treatment series, how difficult or easy did you find it to follow a vegetarian whole food diet?

Difficult	Rather difficult	Neither difficult nor easy	Rather easy	Easy
1	2	3	4	5

6) Would you consider continuing to eat vegetarian food for some days in the future?

Yes	No

a) If so, how often will you eat a vegetarian diet in the future?

Less than 1 day per week	1-2 days per week	3-4 days per week	5-6 days per week	Everyday
1	2	3	4	5

7) How often have you managed to keep your sugar consumption low? (no refined sugar or sweeteners)

Never	Less than 1 day per week	1-2 days per week	3-4 days per week	5-6 days per week	Everyday
1	2	3	4	5	6

Questions concerning nutrition Part 2

8) Did you have any experience with fasting prior to chemotherapy?

Yes	No

9) How often did you fast for 60-72 h during the first 4 chemotherapy appointments?

Never	On one chemotherapy appointments	On two chemotherapy appointments	On three chemotherapy appointments	Each time
1	2	3	4	5

a) If you have not fasted for 60-72 hours on one or more appointments, how many hours did you fast on average?

0 h	1-24 h	25-48 h	49-60 h	> 60 h
1	2	3	4	5

10) In case question 9 was not answered with 'every time':
Where did you experience difficulties?

11) During the approximately 6 months of chemotherapy, how often did you abstain from food overnight?

	Never	1-2 days per week	3-4 days per week	5-6 days per week	Everyday
Abstaining from food for at least 14 h overnight	☐	☐	☐	☐	☐
Abstaining from food for 15 h or longer overnight	☐	☐	☐	☐	☐

12) What was your main diet between chemotherapy appointments?
Several answers are possible

**omnivore** (animal and plant-based foods)	**pescetarian** (vegetarian diet + fish)	**vegetarian wholefood** (plant-based foods, low-fat sour milk products)	**vegetarian** (plant-based foods, eggs, milk and dairy products)	**vegan** (plant-based foods)

13) During the days of fasting, did you consume any food other than juice and broth?

**Yes**	**No**

a) If yes, how often within the first 4 chemotherapy appointments?

**On one chemotherapy appointments**	**On two chemotherapy appointments**	**On three chemotherapy appointments**	**Each time**
**1**	**2**	**3**	**4**

b) How often in the 60 - 72 h around a chemotherapy appointment?

**one meal in all three days**	**two meals in all three days**	**one meal per day**	**two meals per day**	**three meals per day**
**1**	**2**	**3**	**4**	**5**

c) What kind of food did you eat?

Retrospective

14) How did you feel during the days of fasting?
Several answers are possible.

Exhausted	Happy	Tired	Hungry	Satisfied	Normal	In balance	Other:
☐	☐	☐	☐	☐	☐	☐	

15) Did you do any moderate exercise or sports during the approximately 6 months of chemotherapy?

Yes	No

a) If so, how has the vegan diet changed your exercise habits?

Moved a lot less	Moved less	No change	Moved more	Moved much more
1	2	3	4	5

16) Has your quality of life deteriorated or improved due to fasting?

Deteriorated	Rather deteriorated	Constant	Rather improved	Improved
1	2	3	4	5

17) Do you have the impression that fasting in your case has led to a better tolerance of chemotherapy compared to other patients?

No	Little	Average	Pretty much	Very much
1	2	3	4	5

Suggestions for improvement

18) Would you recommend fasting to a friend suffering from breast or ovarian cancer?

No	Rather no	Maybe	Very likely	In any case
1	2	3	4	5

19) Did you have the feeling that you were adequately looked after and supported by doctors / nutritionists / research assistants?

Yes	No

a) In case not, what were you missing?

20) Did you feel that you were adequately looked after and supported by family and friends?

Yes	No

a) If so, did family or friends join in with the fasting?

No	Yes, less than 24 h on average	Yes, on average 24 h	Yes, on average 48 h	Yes, on average 60 h and longer
1	2	3	4	5

b) Did family or friends also eat a vegetarian diet between chemotherapy appointments?

No	Less than 1 day per week	1-2 days per week	3-4 days per week	Yes, always	Other diet:
1	2	3	4	5	

21) Are there any changes you would like us to consider in terms of fasting accompanying chemotherapy that would make fasting easier to accomplish and more practicable?	
**Yes**	**No**
a) If so, please describe them.	

22) Do you have further suggestions for improvement or tips?	
**Yes**	**No**
a) If so, please describe them.	

Questionnaire for dietary intervention vegan diet accompanying chemotherapy

Questions concerning nutrition Part 1

1) The term vegan describes the consumption of vegetable food as well as the simultaneous renouncement of all food of animal origin, like meat, fish, milk, eggs, and honey.

How did you enjoy vegan food?

Not at all	Little	Neither enjoyed nor not enjoyed	Fairly	Very much
1	2	3	4	5

2) How difficult or easy was the preparation of vegan food for you?

Difficult	Rather difficult	Neither difficult nor easy	Rather easy	Easy
1	2	3	4	5

3) Have you had any trouble eating a vegan diet?

No	Little	Average	Pretty much	Very much
1	2	3	4	5

4) Can you imagine following a vegan diet again in the future?

Yes	No

a) If so, how often will you follow a vegan diet in the future?

Less than 1 day per week	1-2 days per week	3-4 days per week	5-6 days per week	Everyday
1	2	3	4	5

A vegetarian wholefood diet means the consumption of vegetable foods and low-fat sour milk products (e.g. yoghurt and soured milk with 1.5% fat or low-fat hard cheese) and the simultaneous avoidance of meat, fish, eggs and other dairy products.

5) In comparison to your previous diet before the chemotherapy treatment series, how difficult or easy did you find it to follow a vegetarian whole food diet?

Difficult	Rather difficult	Neither difficult nor easy	Rather easy	Easy
1	2	3	4	5

6) Would you consider continuing to eat vegetarian food for some days in the future?

Yes	No

a) If so, how often will you eat a vegetarian diet in the future?

Less than 1 day per week	1-2 days per week	3-4 days per week	5-6 days per week	Everyday
1	2	3	4	5

Questions concerning nutrition Part 2

7) Did you have any experience with a vegan diet before the chemotherapy treatment?

Yes	No

8) How often did you follow a vegan diet for 60 to 72 hours during the first 4 chemotherapy sessions?

Never	On one chemotherapy appointments	On two chemotherapy appointments	On three chemotherapy appointments	Each time
1	2	3	4	5

a) If you did not follow a vegan diet for 60-72 hours on one or more appointments, how many hours on average did you follow a vegan diet on this or these appointments?

0 h	1-24 h	25-48 h	49-60 h	> 60 h
1	2	3	4	5

b) Remember a chemotherapy appointment when you weren't on a vegan diet for 60 to 72 h...

For which meals did you eat animal foods such as meat, fish, dairy products or eggs?
Several answers are possible.

Breakfast	Snack	Lunch	Snack	Dinner	Further meal:
1	2	3	4	5	

By <u>sugar reduction</u> we mean the renunciation of quickly absorbable carbohydrates such as refined sugar as well as the renunciation of sweeteners.

9) In addition to the vegan diet, how often did you maintain a sugar reduction over the period of 60-72 h within the first 4 chemotherapy appointments?

Never	On one chemotherapy appointments	On two chemotherapy appointments	On three chemotherapy appointments	Each time
1	2	3	4	5

a) If you have not reduced your sugar consumption on one or more appointments for 60 to 72 hours, how many hours on average did you reduce it?

0 h	1-24 h	25-48 h	49-60 h	> 60 h
1	2	3	4	5

b) Remember a chemotherapy appointment where you did not reduce sugar in your diet for 60-72 hours...

At which meals did you consume sugary food and beverages?

Breakfast	Snack	Lunch	Snack	Dinner	Further meal:
**1**	**2**	**3**	**4**	**5**	

10) In case question 8 was not answered with 'every time':
Where did you experience difficulties?

a) In case question 9 was not answered with 'every time':
Where did you experience difficulties?

11) Fasting was never prescribed at any time in your intervention group. Nevertheless, during the first 4 chemotherapy appointments, did you fast on one or more appointments within the 60-72 hours or simply did not eat anything?

No	Up to 14 h	Up to 24 h	Up to 48 h	Up to 72 h
**1**	**2**	**3**	**4**	**5**

a) If you fasted for up to 24 hours, was that the day the chemotherapy took place?

Yes	No

12) At no time in your intervention group was overnight food abstinence a requirement. Did you nevertheless abstain from food overnight during the approximately 6 months of chemotherapy?

	**Never**	**1-2 days per week**	**3-4 days per week**	**5-6 days per week**	**Everyday**
Abstaining from food for at least 14 h overnight	☐	☐	☐	☐	☐
Abstaining from food for 15 h or longer overnight	☐	☐	☐	☐	☐

13) What was your main diet between chemotherapy appointments?
Several answers are possible

**omnivore** (animal and plant-based foods)	**pescetarian** (vegetarian diet + fish)	**vegetarian wholefood** (plant-based foods, low-fat sour milk products)	**vegetarian** (plant-based foods, eggs, milk and dairy products)	**vegan** (plant-based foods)

a) How often have you managed to keep your sugar consumption low between chemotherapy appointments? (no refined sugar or sweeteners)

**Never**	**Less than 1 day per week**	**1-2 days per week**	**3-4 days per week**	**5-6 days per week**	**Everyday**
**1**	**2**	**3**	**4**	**5**	**6**

Retrospective

14) How did you feel during the vegan diet?
Several answers are possible.

Exhausted	Happy	Tired	Hungry	Satisfied	Normal	In balance	Other:
☐	☐	☐	☐	☐	☐	☐	

15) Did you do any moderate exercise or sports during the approximately 6 months of chemotherapy?

**Yes**	**No**

a) If so, how has the vegan diet changed your exercise habits?

**Moved a lot less**	**Moved less**	**No change**	**Moved more**	**Moved much more**
**1**	**2**	**3**	**4**	**5**

16) Has your quality of life deteriorated or improved due to the vegan diet?

**Deteriorated**	**Rather deteriorated**	**Constant**	**Rather improved**	**Improved**
**1**	**2**	**3**	**4**	**5**

17) Do you have the impression that a vegan diet in combination with a sugar reduction in your case has led to a better tolerance of chemotherapy compared to other patients?

**No**	**Little**	**Average**	**Pretty much**	**Very much**
**1**	**2**	**3**	**4**	**5**

Suggestions for improvement

18) Would you recommend the vegan diet in combination with a sugar reduction to a friend suffering from breast or ovarian cancer?

No	Rather no	Maybe	Very likely	In any case
1	2	3	4	5

19) Did you have the feeling that you were adequately looked after and supported by doctors / nutritionists / research assistants?

Yes	No

a) In case not, what were you missing?

20) Did you feel that you were adequately looked after and supported by family and friends?

Yes	No

a) If so, did your family or friends also follow a vegan diet?

No	Yes, less than 24 h on average	Yes, on average 24 h	Yes, on average 48 h	Yes, on average 60 h and longer
1	2	3	4	5

b) Did family or friends also eat a vegetarian diet between chemotherapy appointments?

No	Less than 1 day per week	1-2 days per week	3-4 days per week	Yes, always	Other diet:
1	2	3	4	5	

21) Are there any changes you would like us to consider in terms of vegan nutrition in combination with a sugar reduction concomitant to chemotherapy that would make the dietary change easier to implement and more practical?

Yes	No

a) If so, please describe them.

22) Do you have further suggestions for improvement or tips?

Yes	No

a) If so, please describe them.
